# A Comprehensive Analysis of the Effectiveness of a Water-Based Extraction Method in Cement Bypass Dust Valorization

**DOI:** 10.3390/ma18204668

**Published:** 2025-10-11

**Authors:** Karolina Wojtacha-Rychter, Magdalena Król, Jakub Dechnik

**Affiliations:** 1 Central Mining Institute—National Research Institute, Pl. Gwarków 1, 40-166 Katowice, Poland; 2 Faculty of Materials Science and Ceramics, AGH University of Krakow, 30 Mickiewicza Av., 30-059 Krakow, Poland; mkrol@agh.edu.pl (M.K.); jdechnik@agh.edu.pl (J.D.)

**Keywords:** bypass dust, chlorine, cement industry, leaching, XRD, SEM, IR spectroscopy

## Abstract

The solid by-product from cement kiln gas installations, known as cement bypass dust (CBPD), is rich in chlorides, which limits the reuse of materials in cement. In this study, three types of CBPD were subjected to an extraction process to obtain a low-chlorine waste material. The relationships between the process parameters, including extraction time (1, 2, 5, 10, and 30 min), temperature (21, 45, and 90 °C), and extraction efficiency, were investigated. The chlorine removal efficiency ranged from 70% to 90%, with the optimal time and temperature identified as 1 min and 21 °C, respectively. Furthermore, a comprehensive characterization of CBPD was conducted before and after the extraction process using X-ray diffraction (XRD), X-ray fluorescence (XRF), scanning electron microscopy (SEM), and Fourier transform infrared spectroscopy (FT-IR); an approach not yet extensively reported in the literature. The results demonstrated that chloride removal corresponded to an increase in concentrations of Ca, Al, Si, Mg, and Fe oxides in the solid residue. For CBPD samples with initial chloride contents of 13.65% and 15.43%, calcium content in the residue increased by approximately 40%. No linear and predictable relationship was observed between the leaching time or temperature and the release of metals in the solid residue.

## 1. Introduction

Many cement plants have increasingly turned to alternative fuels (AF) as a source of thermal energy [1] to promote a circular economy by maximizing resource recovery from waste. An approach aligns with the EU’s waste policy [2]. In European countries and parts of North America, the thermal substitution rate (TSR) ranges from 20% to 90%, whereas in China’s cement sector, the average TSR was only around 5% in 2023 [3]. The highest reported AF usage rate of 98.7% was achieved by the Vicat plant at Vigier in Switzerland [4]. However, the growing use of solid alternative fuels presents new operational challenges, particularly the increased generation of chlorine-rich cement bypass dust (CBPD).

Chlorine is typically present in alternative fuels either as inorganic chloride substances or in various organic chlorinated forms. Organic chlorine compounds are often generated by plastic waste combustion, while inorganic chlorides primarily originate from food and kitchen waste, which contains alkali metal salts such as NaCl and KCl [5]. During AF incineration, chlorine is released in the form of HCl, alkali metal chlorides, and/or heavy metal chlorides. At temperatures of up to ~400 °C, HCl can decompose via the Deacon reaction, generating molecular chlorine [6], which reacts with alkali metals to form low-melting eutectic salt mixtures. These salts tend to condense in the cooler zones of the preheater tower, resulting in the adhesion of kiln feed particles and the formation of deposits that may block gas and material flow in the kiln system [7,8].

To address the issue of alkali and chlorine build-up in kiln circuits, cement plants implement bypass systems [9,10] that remove excess chlorine-laden fine particles from the process and store them in silos. In the cement industry, the terms Cement Kiln Dust (CKD) and Cement Bypass Dust (CBPD) are sometimes used interchangeably, although they refer to related but distinct materials. CKD generally denotes the fine particulate matter that is carried out in a stream of the exhaust gas from the rotary kiln and cyclone preheaters, and is collected in bag filters. In contrast, CBPD specifically refers to the dust collected from the chlorine bypass system located between the kiln and preheaters [11]. Both CKD and CBPD contain fine raw materials used in kiln feed, partially some materials calcined by heat, clinker, and volatile compounds, such as alkalis, chlorides, and sulfur [12]. Due to differences in the exhaust gas removal systems of clinker kilns, CBPD usually exhibits higher concentrations of alkalis and chlorides compared to general CKD, making its management more challenging.

In this study, the term CBPD is used exclusively to describe the material obtained from the chlorine bypass system, unless otherwise stated. The chemical composition of CBPD is similar to that of partially clinkered raw material, and it may retain some hydraulic activity. These characteristics have led to various propositions for applying CBPD in building materials, as documented in scientific literature [13,14,15]. However, one of the major barriers to repurposing CBPD in cement and concrete is its high chlorine content, which can reach up to 15% by weight [16,17,18,19]. Chlorides are known to promote corrosion of steel reinforcement in concrete, necessitating the strict limitation of their content [20]. According to standard EN 197-1 [21], the maximum permissible chloride content in cement is 0.1% by mass. Therefore, effective chloride removal methods are essential in order to enable the safe repurposing of CBPD in building materials.

Consequently, some researchers have explored the other potential repurposing pathways, such as soil stabilization, wastewater treatment, ceramic and mine backfill, for chlorine-rich cement kiln by-products and CKD beyond their direct application as cement additives [12,22]. For example, Al-Rawas [23] used cement bypass dust in soil stabilization, Taha et al. [24] incorporated it as filler in asphalt concrete mixtures, while Ata et al. [25] applied it in backfilling slurry cutoff walls. El Zayat [26] studied the potential of CKD to adsorb heavy metal cations from wastewater. Other studies [27,28,29] investigated the use of bypass dust in the development of alkali-activated materials.

An alternative approach is to convert CBPD into a more valuable resource by removing its undesirable components (primarily chlorides) through leaching. The simplest method involves dissolving the chlorine compounds using a solvent. Daous [30] investigated alkali leaching from Cement Kiln Dust (CKD) with low chloride concentrations using water and aqueous calcium nitrate solutions at temperatures ranging from 30 to 80 °C and liquid-to-solid weight ratios of 3:1, 5:1, and 10:1, to produce potassium-nitrogen fertilizers. Seo et al. [31] demonstrated that the leaching time and solvent type both significantly affect the chloride and calcium removal efficiency from the solid residue. Another approach developed by Wang et al. [32] combined bypass dust washing with salt extraction from the leachate, using steam from waste-heat power generation as a heat source. Choi et al. [33] employed water and sodium hydroxide under ambient conditions to separate potassium chloride from cement kiln dust, reporting an improvement in mortar compressive strength by over 20% when an activator derived from the cement dust was added. Baran et al. [34] demonstrated that the residue obtained after extracting chlorine and potassium could be repurposed as a raw material in Portland clinker production. Their calculations indicated that replacing 87.07% of the raw mix with the extraction residue containing 58.52% CaO could reduce CO_2_ emissions by 407.6 kg per Mg of clinker. The cement produced from such clinker exhibited superior mechanical properties compared to traditional Portland cement, attributed to a higher alite content. Lee et al. [35] proposed selective calcium and potassium chloride extraction using water and hydrochloric acid, and found that aqueous extraction increases the solid residue pore volume.

In light of these findings, the present study proposes a water-based extraction process as an efficient, environmentally friendly, and cost-effective method of transforming CBPD into a more valuable material. The study investigates the influence of key process parameters, such as extraction time, temperature, and initial chemical composition of the dust, on the chloride leaching efficiency. To our knowledge, such an in-depth analytical approach has not yet been reported in the literature. In addition to evaluating chloride removal, the study provides mechanistic insights into the changes occurring in the solid residue, including the formation of secondary phases such as portlandite and ettringite, as well as a relative increase in the concentration of metal oxides (Ca, Al, Si, Mg, Fe). Therefore, the novelty and added value of our study lie in expanding the current understanding of cement industry by-product valorization, contributing to resource efficiency, supporting the principles of circular economy, and facilitating the industrial-scale repurposing of cement kiln by-products.

## 2. Materials and Methods

### 2.1. Materials

Three fine-grained, highly alkaline by-product, cement bypass dusts (CBPD), were subjected to a water-based extraction process. All CBPD samples were collected from the chlorine bypass dust installation applied between the kiln and cyclone preheaters in three different local cement plants. Samples were stored in tightly sealed bags for no longer than one month before analysis.

### 2.2. Experimental Procedure

The leaching experiments were conducted in glass beakers placed directly on a magnetic stirrer equipped with a heating functionality. The CBPD samples were mixed with demineralized water at a solid-to-liquid ratio of 1 g per 10 mL. A constant stirring speed of 500 rpm was maintained throughout the process. To investigate the effect of temperature on chloride leaching, three experiments were carried out at 21 °C, 45 °C and 90 °C, each with a fixed leaching duration of 30 min. Once the solvent reached the target temperature, the CBPD powder was added to the stirred water. Based on the results of these tests, 21 °C was selected as the optimal temperature for further experiments. The effect of leaching time on chloride removal was then evaluated at 21 °C for all the CBPD samples. The leaching durations were set at 1, 2, 5, 10 and 30 min. After each specified time interval, the suspensions were vacuum-filtered to separate the solid and liquid phases. The solid leaching residues were then dried in an oven at 100 ± 5 °C overnight. The mass of the dried residues was recorded, showing that approximately 74%, 73%, and 99% of the initial mass was retained for samples A, B, and C, respectively, with variations due to leaching time and temperature within ±2%. All the dried leaching residues were subjected to chemical, mineralogical and microstructural analyses. The chloride ion concentration in the filtrates was determined using Mohr’s titration method [36].

The chloride ion leaching efficiency from bypass dust was estimated using the following Equation (1) [35]:

EE = (C_in solution_ × V_solution_)/(C_in solid_ × M_solid_) × 100% (1)

where EE is the extraction efficiency (%), C_in solution_ is the measured chlorine concentration in the solution (g/L), C_in solid_ is the chlorine percentage in the bypass dust (%), V_solution_ is the solvent volume (L), M_solid_ is the bypass dust mass (g). The chloride ions were determined by titration in leachates, according to standard PN ISO 9297:1994 [36], and by X-ray fluorescence (XRF) in solid leaching residue.

The effect of the leaching conditions on the selected metal concentrations in cement bypass dust was evaluated using Equation (2), expressed as percent change (PC). PC indicates the relative increase or decrease in concentration compared to the initial value. A positive value denotes an increase, while a negative value indicates a decrease.

PC = (Me_final_ × Me_initial_)/(Me_initial_) × 100% (2)

where PC is percent change (%), Me_final_ is the measured metal concentration in solid residue (%) after leaching, Me_initial_ is the metal percentage in the bypass dust (raw material) (%). All experimental data are averages of the three measurements.

### 2.3. Methods

#### 2.3.1. X-Ray Diffraction (XRD)

A Philips X’Pert Pro diffractometer (PANalytical, Malvern, UK) was used to identify the individual tested sample phases. A copper (CuKα) anode lamp with a linear focus was used to obtain a monochromatic beam. Diffraction patterns were recorded in the 2θ range of 5–60°. Prior to analysis, the samples were ground into fine powder to ensure homogeneity and facilitate accurate phase identification. Phase analysis was conducted using the Crystallography Open Database (COD) and the Match! 4 software for pattern matching and interpretation.

#### 2.3.2. X-Ray Fluorescence Spectroscopy (XRF)

Elemental composition was determined using an Axios mAX 4 kW wavelength dispersive X-ray fluorescence (WD-XRF) spectrometer (PANalytical, Malvern, UK). The spectrometer was equipped with a rhodium (Rh) source. The XRF method makes it possible to detect elements from beryllium to uranium at various concentration levels using both single and complex crystal analyzers. For the analyses, the samples were prepared as pressed powder pellets. All analyses were carried out in standard mode using the Omnian software package (PANalytical, Malvern, UK).

#### 2.3.3. Fourier-Transform Infrared Spectroscopy (FT-IR)

Infrared absorption spectra were collected using a Bruker Verteks 70V vacuum spectrometer (Bruker, Billerica, MA, USA). Measurements were performed in the mid-infrared region. A total of 128 scans were conducted for each sample to improve signal-to-noise ratio. Spectra were recorded in absorbance mode with a spectral resolution of 4 cm^−1^. The obtained spectra were linear baseline-fitted and min-max normalized using OPUS 7.2 software to ensure accurate absorption band interpretation.

#### 2.3.4. Scanning Electron Microscopy (SEM)

A microstructural analysis was conducted using a Phenom XL (PIK Instruments, Waltham, MA, USA) scanning electron microscope. This is a non-destructive method with minimal sample preparation required. The tested materials were affixed to carbon adhesive tape and coated with an 8 nm layer of gold using a sputter coater to enhance conductivity. Imaging was performed at an accelerating voltage of 10 kV. Additionally, the microscope was equipped with an energy-dispersive X-ray spectroscopy (EDS) detector, enabling the semi-quantitative elemental analysis of selected micro-areas.

## 3. Results and Discussion

### 3.1. Starting Material Characterization

The chemical compositions of the CBPD samples, expressed as oxide contents, are presented in Table 1.

**Figure 1:**
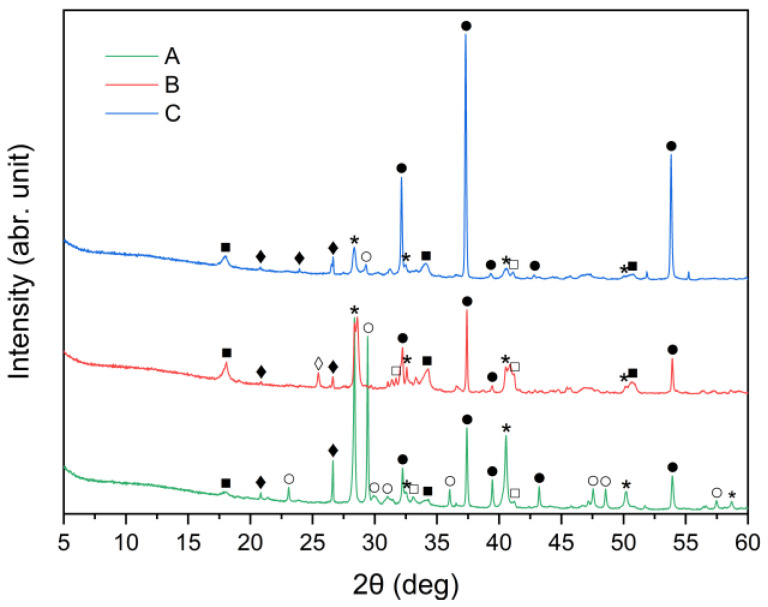
X-ray diffraction patterns of sample A, B, and C (⁎—sylvite (KCl); ●—lime (CaO); ○—calcite (CaCO_3_); ■—portlandite (Ca(OH)_2_); □—alite (Ca_3_SiO_5_); ♦—quartz (SiO_2_); ◊—anhydrite (CaSO_4_)).

X-ray diffraction analysis results for the three cement bypass dust samples are presented in Figure 1. The XRD patterns indicate that the dominant crystalline phase in all the samples was sylvite (KCl). The intensity of the peak at 2θ ≈ 28° corresponds to the chlorine content determined by XRF, as provided in Table 1. Calcium oxide (CaO) was present in three different crystalline forms: calcium carbonate (CaCO_3_, calcite), calcium hydroxide (Ca(OH)_2_, portlandite) and free CaO (free-lime). Among these, free CaO was the dominant phase in all three samples. In particular, the XRD patterns for samples A, B and C were primarily characterized by strong peaks corresponding to free CaO. Calcite and portlandite peaks were generally weak, with the exception of sample A, which demonstrated a noticeably higher intensity of the calcite peak compared to samples B and C. The high calcite content in CBPD was also reported in work by Abiad et al. [13]. Calcite may come from unprocessed raw materials. Additionally, sample C exhibited the highest intensity of the portlandite peak, corresponding well to the XRF results presented in Table 1. The quartz (SiO_2_) peak at 2θ ≈ 26° was relatively weak in all the CBPD samples. The obtained mineralogical compositions conform with previously published data [19,27,37].

### 3.2. Effect of Temperature and Contact Time on the Leachability of Metals and Chlorine

Three cement bypass dust samples (A, B, and C), differing in the concentration of metals (Ca, Al, Fe, Mg, Si) and chlorides, were used as raw materials. These samples were subjected to a water-based extraction process to investigate the reduction in chloride content and changes in chemical composition under varying process parameters, specifically temperature and extraction time.

**Figure 2:**
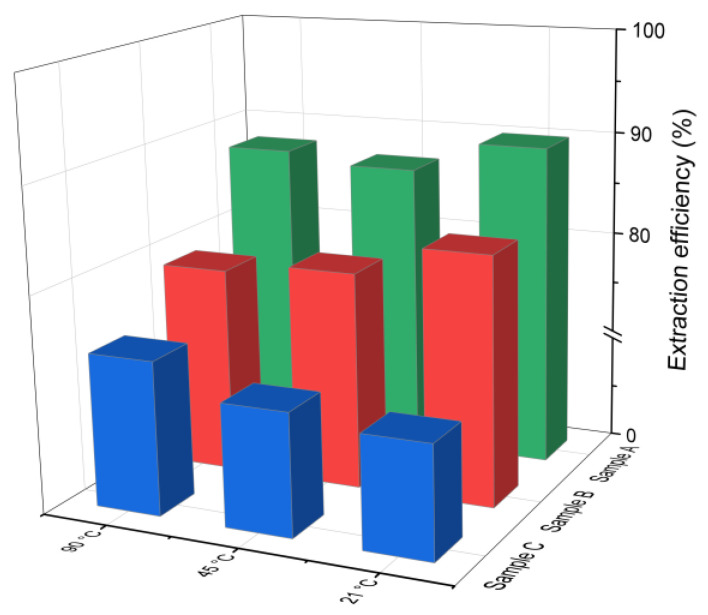
Chloride ion leaching efficiency for samples A, B and C with water as a function of the reaction temperature (using Equation (1)).

The chlorine leaching efficiency (calculated using Equation (1)) for samples A, B and C after 30 min of water-based extraction at different temperatures is presented in Figure 2. As can be observed, effective chlorine removal was achieved even at the lowest tested temperature of 21 °C. The extraction efficiency for samples A and B was higher compared to that of sample C, which had a lower initial chloride content (4.3%). This is in line with findings reported by [38], indicating that higher initial chloride concentrations generally improve the leaching efficiency. No significant differences in the chlorine removal were observed across the tested temperature range. In sample B, the leaching efficiency decreased slightly by 3–4% as the temperature increased from 21 °C to 90 °C, while in sample C, it increased by about 4%. In the case of sample A, the efficiency initially decreased and then increased with temperature. This nonlinear behavior may be related to the strongly alkaline conditions of the leachates (pH 12–13), which can influence the solubility of chlorine-bearing compounds and thus affect the leaching performance.

**Figure 3:**
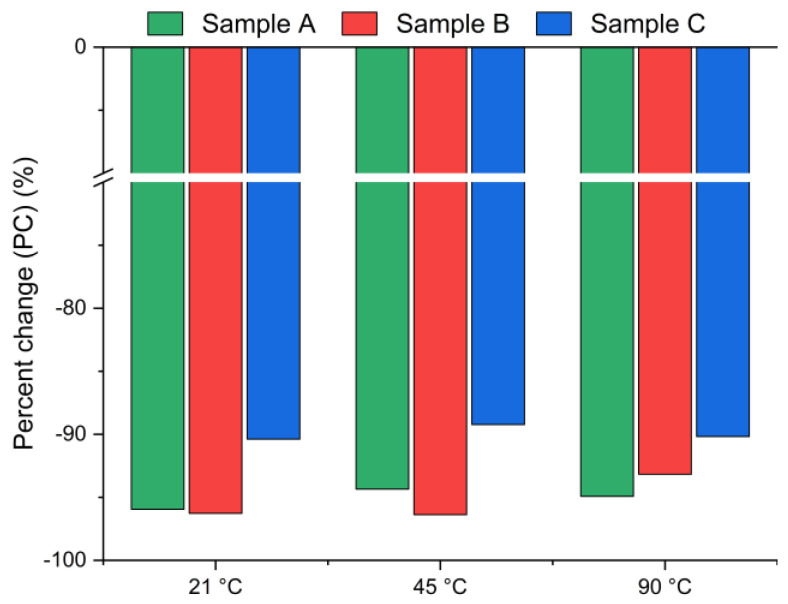
Chloride ion percent change in bypass dust as a function of the reaction temperature (using Equation (2)).

Figure 3 presents the chlorine ion removal efficiency for samples A, B and C calculated using Equation (2), based on the chloride content measured in the solid residue via XRF. A negative value indicates a decrease in chlorine concentration in the solid phase after leaching. Interestingly, the results reveal significantly higher leaching efficiencies (>90%) compared to those presented in Figure 2. The differences between the leaching efficiency values are likely due to the use of different analytical methods: Mohr’s titration for the leachate and XRF for the solid residue. While Mohr’s titration is a sensitive method for determining soluble chlorides, the precision of endpoint detection can be affected by operator subjectivity, coexisting ions, or incomplete precipitation of AgCl, potentially leading to an underestimation of chloride ion concentrations in the solution [39]. The endpoint is determined by visual observation of the color change from yellow to red-brown. The difference that exists between the observed endpoint and the actual chemical equivalence in titration may lead to slight errors in the results [40]. In contrast, XRF quantifies the total elemental composition of the solid residues, including bound or less soluble chlorine forms, and therefore may yield systematically different values. The observed discrepancies highlight the complementary nature of these two techniques: titration provides direct information about the soluble fraction of chlorides, whereas XRF captures the total chlorine content in the solid phase. Taken together, the results consistently confirm that chlorine was effectively removed under the applied conditions.

In other papers [31,41], authors conducted an experimental study on chloride extraction by alternative methods based on the strong acidic and basic solutions (e.g., Ca(NO_3_)_2_, HCl, NaOH). Seo et al. [31] observed that at a liquid to solid ratio of 10, the removal efficiencies of three organic acids was twice as effective as that of distilled water; however, when increasing the ratio to 20, distilled water achieved an efficiency of chloride > 90%. Zhao et al. [42] reported that sodium hydroxide and ethanol both produced a chloride leaching rate of 68.9% in a magnesium hydroxide powder. Youn [43] found that the removal efficiency of potassium chloride from industrial cement by-products was 73% by applying pure water and an HCl solution.

It is important to note that water, as a solvent, is safer, cheaper, and has minimal environmental impact compared to acidic and basic solutions. Thus, considering both performance and energy savings, a process temperature of 21 °C and a time of 30 min can be considered optimal for chlorine removal from Cl-rich CBPD. Under these conditions, the chloride ion content in solid residues was <0.5%. According to EN 197-1: 2012 [21], the chlorine content limit in cement is 0.1%, while the total chlorine content in concrete in Europe is limited by EN 206: 2013 [44] and ranges from 0.1 to 1.0% by weight of cement depending on the type of reinforcement or other embedded metal. An example calculation of total chlorine content in a concrete mixture [45], where 10% of cement is replaced by CBPD is presented in Table 2. Based on results in Table 2, the chloride content by weight of cement is 0.22%. Thus, CBPD content increase by 5% in the concrete mixture will be involved an increase in total chlorine content in concrete by 0.1–0.2% by weight of cement. According to the European Concrete Standard, BS EN 206 1 [44], at 0.22% the concrete chloride class declared would be Cl 0.40, i.e., concrete containing steel reinforcement or other embedded metal. In addition, of course, it would be necessary to carry out the impact of dechlorinated CBPD on the mechanical and durability properties of cement-based materials to fully validate that strength criteria are met.

**Figure 4:**
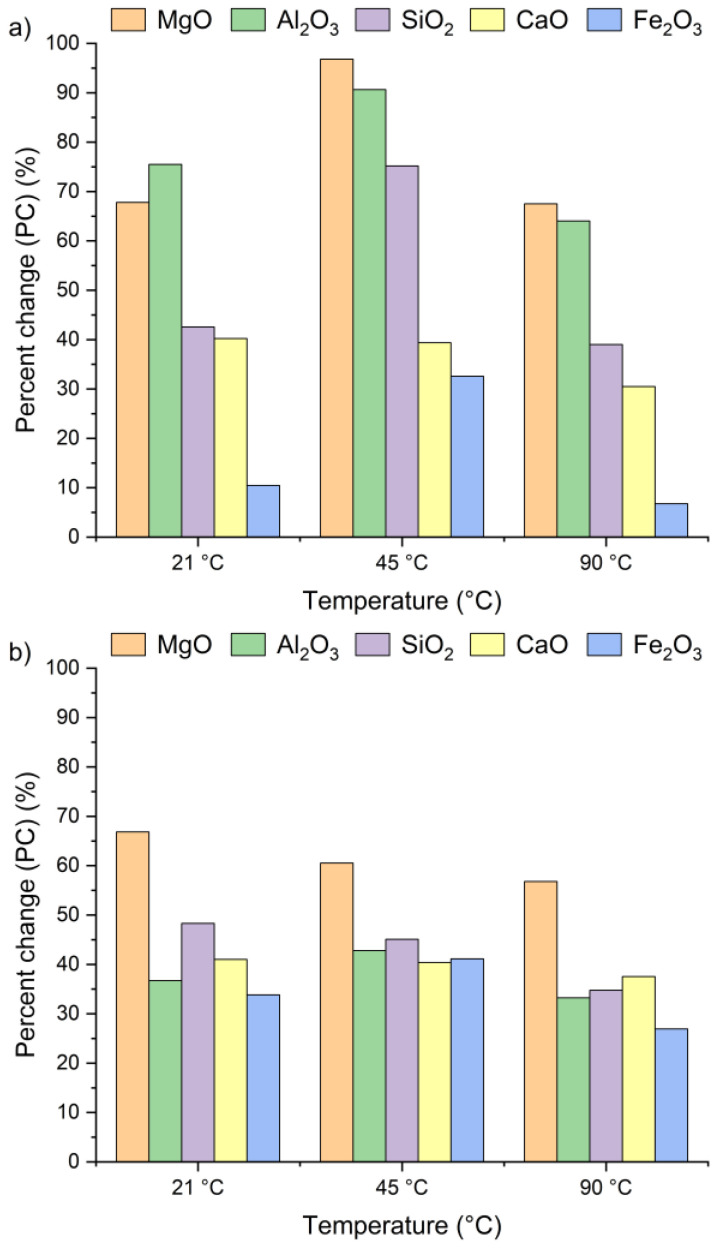

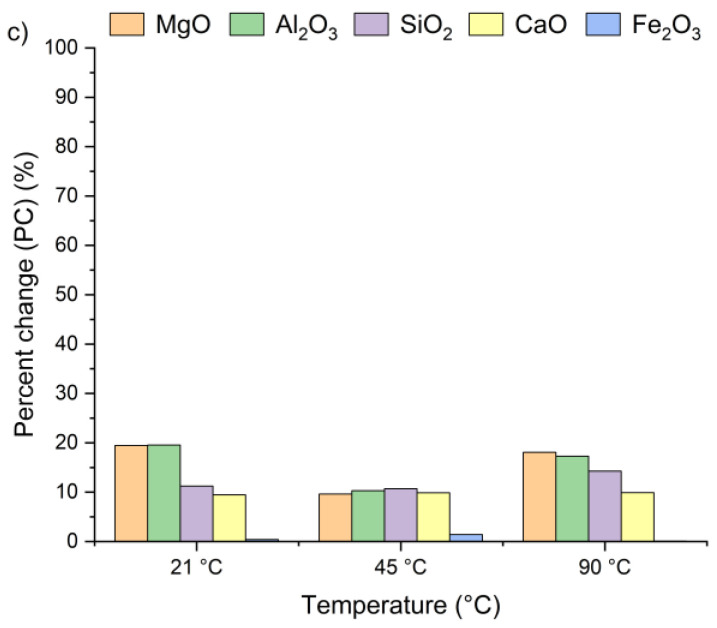
Percent change in Ca, Si, Al, Fe, and Mg oxides in the solid as a function of the reaction temperature (using Equation (2)): (**a**) sample A, (**b**) sample B, (**c**) sample C.

Figure 4 presents the effect of leaching temperature on the change in selected metal oxide concentrations in samples A, B, and C. The results are expressed as percent change (PC), representing the relative difference in metal oxide content in the solid residue after the leaching process (calculated using Equation (2)).

The positive percent change values for all the samples indicate an increase in metal oxide concentrations in the solid residues compared to the original raw material. In the case of samples A and B, no clear linear relationship was observed between the temperature and the concentrations of Al_2_O_3_, SiO_2_, MgO, and Fe_2_O_3_ in solid residues. As depicted in Figure 4a,b, the concentrations of SiO_2_, Al_2_O_3_, and Fe_2_O_3_ initially increased from 21 °C to 45 °C and then decreased as the temperature rose to 90 °C. An exception was observed for CaO, whose concentration in samples A and B slightly decreased, by ~1–3%, together with increasing temperature. This suggests that the highest CaO content in the solid residue can be obtained at 21 °C. The decrease in metal oxide concentrations at the highest temperature may be related not only to changes in solubility equilibria but also to the stability of secondary phases under leaching conditions. Although the leachates remained strongly alkaline (pH 12–13) across all temperatures, higher temperatures could shift the solubility equilibria of certain metal hydroxides (e.g., Mg(OH)_2_, Fe(OH)_3_), promoting their precipitation and retention in the solid phase [42,47,48]. Moreover, the increase in oxide concentration in solid residues may also be attributed to the reprecipitation of metal ions as hydroxides, or their incorporation into newly formed secondary phases such as ettringite or portlandite. These processes are favored by high pH conditions and the presence of abundant calcium ions, which facilitate the immobilization of Al^3+^, Mg^3+^ or Fe^3+^. Among all the analyzed oxides, MgO reached the highest PC, while Fe_2_O_3_ exhibited the lowest. In sample A, the percent change in MgO ranged from 67% to 97% and in sample B from 51% to 67%, depending on temperature. This indicates a nearly twofold increase in MgO concentration in the solid residue. Interestingly, the concentrations of metal oxides in sample C remained largely unaffected by temperature variations and demonstrated considerably lower percent change values compared to samples A and B. This behavior may be linked to the lower chloride content in sample C (see Table 1). The apparent increase in Ca, Al, Si, Mg, and Fe oxides observed in samples A and B after chloride removal is primarily a relative effect caused by the dissolution of highly soluble salts, such as KCl, NaCl, and partially sulfates. This selective leaching reduces the overall solid mass while leaving the main silicate and aluminate phases largely unaffected, which results in an apparent enrichment of these oxides in the residue. Such concentration effects are typical in alkaline leaching systems. The pH of wet CBPD is strongly basic (12–14) [49], due to the presence of alkali compounds that dissociate and release hydroxide ions (OH^−^). In this environment, the dominant oxides remain stable and largely insoluble [50,51]. In contrast, sample C exhibited only minor compositional changes, likely reflecting that its metals were already present in stable, insoluble forms within the raw material.

### 3.3. Effect of Leaching Time

**Figure 5:**
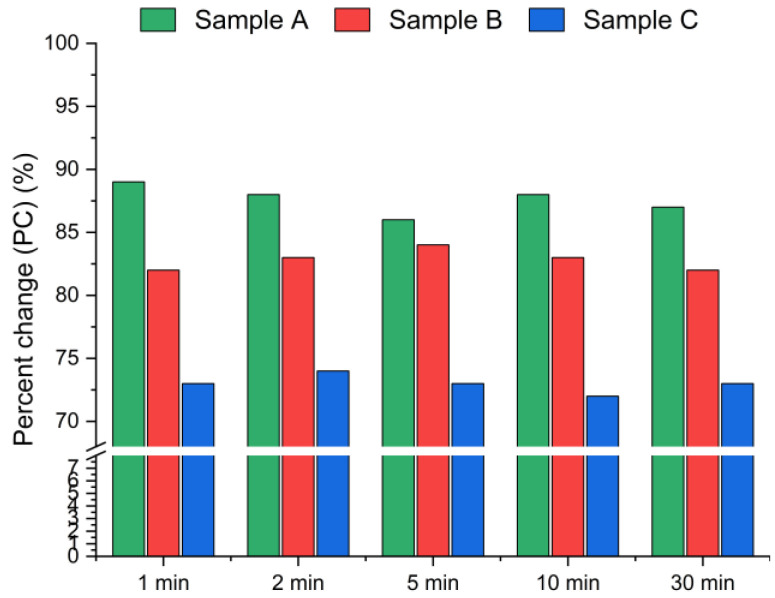
Chloride ion leaching efficiency for samples A, B and C with water as a function of the reaction time (using Equation (1)).

**Figure 6:**
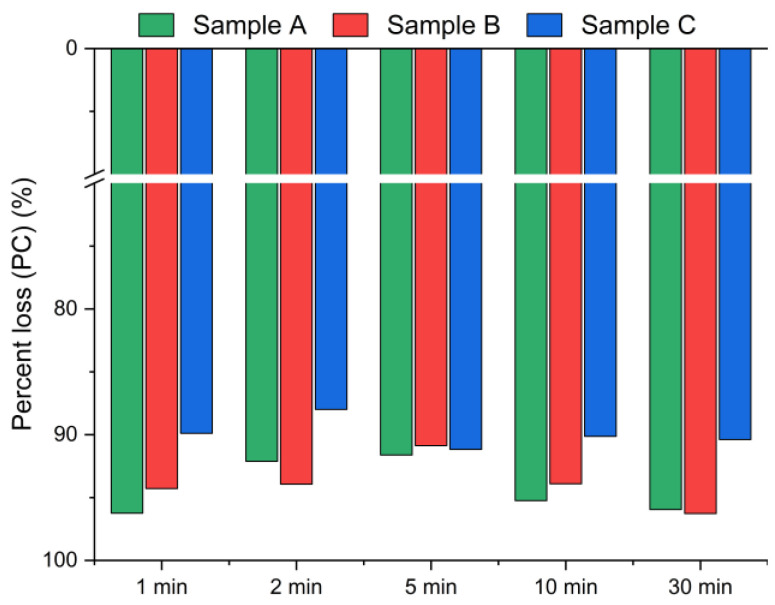
Chloride ion percent change in bypass dust as a function of the reaction time (using Equation (2)).

The results presented in Figure 5 and Figure 6 display the effect of leaching time (1 min, 2 min, 5 min, 10 min and 30 min) on the chlorine removal efficiency at a constant temperature of 21 °C and a fixed water-to-solid ratio of 10:1.

Figure 5 presents the chlorine removal efficiencies calculated based on the chloride concentration in the leachate, according to Equation (1), while Figure 6 presents the results calculated using Equation (2), based on the chlorine content in the solid residues obtained from the XRF analysis. The data indicate that the leaching process was the most effective within the first min of water contact. The chlorine removal efficiency reached 70–90% after just 1 min (Figure 5), and extending the leaching time further resulted in no significant improvement in chlorine removal. The best effects, i.e., the highest chlorine removal efficiency, were observed for sample A. In contrast, sample C exhibited the lowest efficiency, with just above 70% of chlorine removed, regardless of the leaching duration. These findings suggest that chlorine present in CBPD is predominantly associated with highly soluble salts, such as KCl and NaCl, which dissolve almost instantaneously upon contact with water.

Based on the XRF analysis results in Figure 6, the chlorine content in the bypass dust decreased by over 93% for samples A and B, and by ~90% for sample C within the first min of leaching.

**Figure 7:**
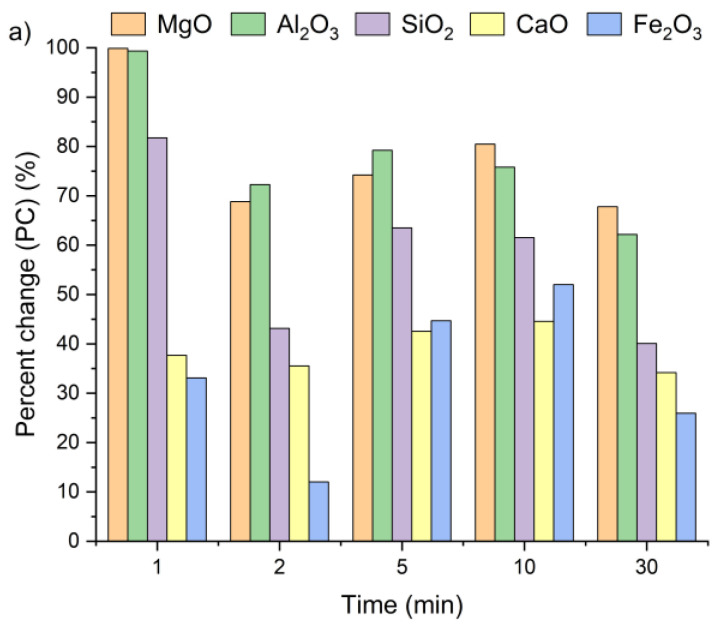

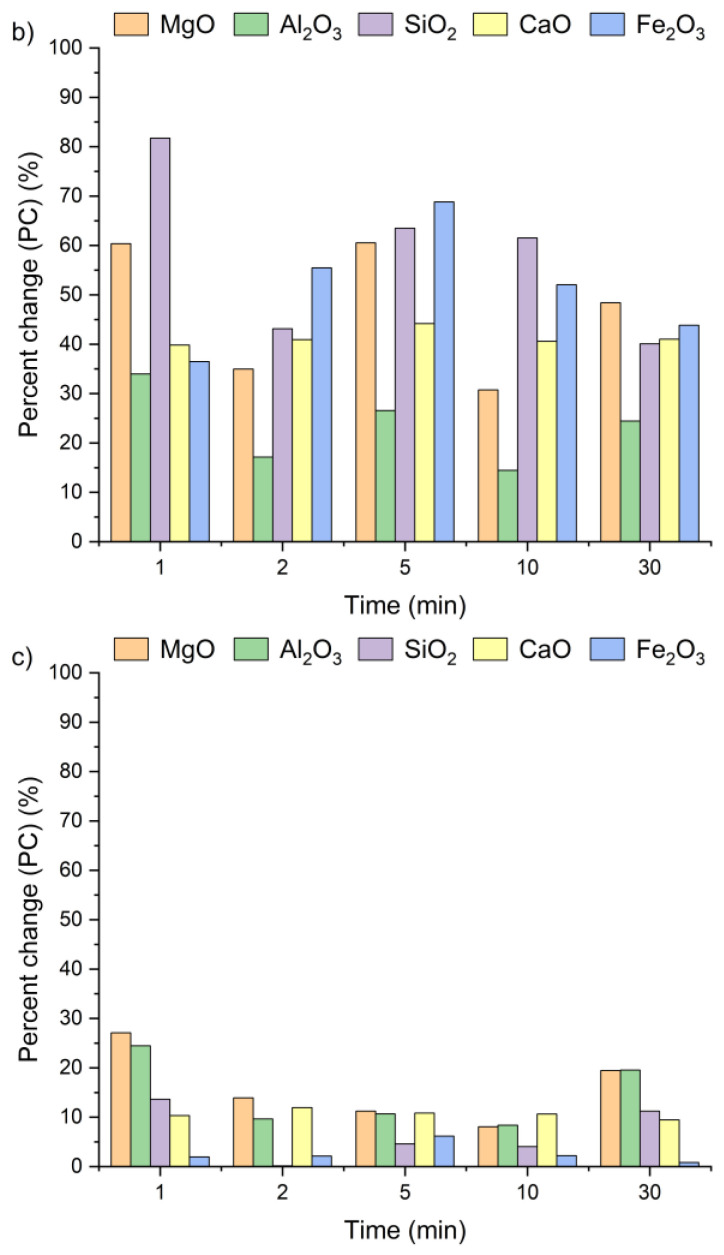
Ca, Si, Al, Fe, and Mg ion percent change in (**a**) sample A, (**b**) sample B, and (**c**) sample C as a function of the reaction time (using Equation (2)).

Figure 7 presents the changes in oxide concentrations in samples A, B and C after leaching for 1, 2, 5, 10, and 30 min, expressed as a percent change. Positive PC values indicate an increase in selected oxide content in the solid residues of all the tested samples, regardless of leaching time. Interestingly, a visible increase in oxide concentrations was already observed after the first minute. It can be clearly witnessed that extending the leaching time had only a minor effect on the calcium concentration. After 30 min of leaching, the CaO content in the solid residues was only 13% higher compared to the values recorded after 1 min. The highest calcium content relative to the initial concentration (Table 1) was detected within a 5–10 min interval. These findings are in line with results previously reported by Lee et al. [35] and Seo et al. [31].

The highest SiO_2_ concentrations in the solid residues of samples A and C were observed after 1 min of leaching, while for sample B, the peak occurred at 5 min. The highest Al_2_O_3_ and MgO concentrations were reached after 1 min for all the tested samples, whereas the maximum Fe_2_O_3_ content was obtained after 5 min of leaching. Based on these observations, a 5 min leaching time appears sufficient to achieve the highest concentrations of metal oxides in the solid residue.

### 3.4. Effect of Leaching Conditions on the Mineralogical Composition and Surface Chemistry

**Figure 8:**
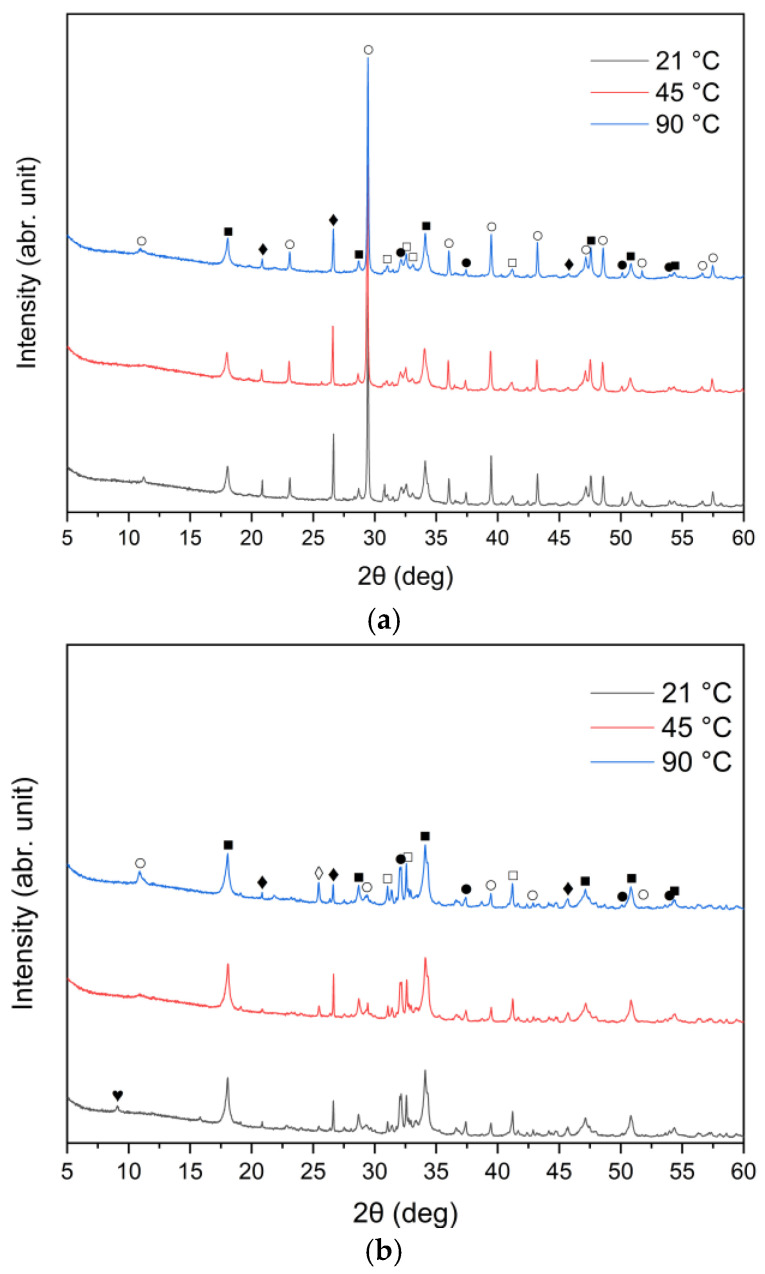

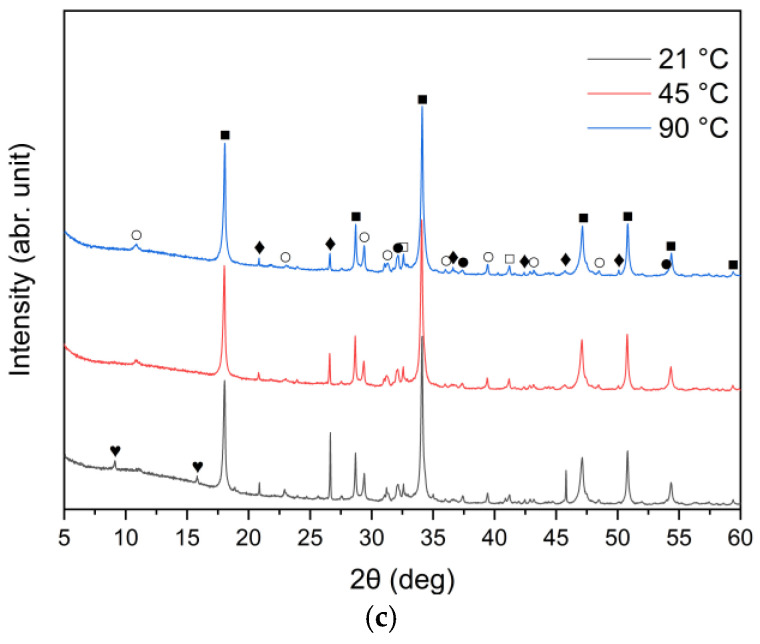
X-ray diffraction patterns of solid leaching residues after 30 min leaching for (**a**) sample A, (**b**) sample B, and (**c**) sample C at different temperatures (●—lime (CaO); ○—calcite (CaCO_3_); ■—portlandite (Ca(OH)_2_); □—alite (Ca_3_SiO_5_); ♦—quartz (SiO_2_); ◊—anhydrite (CaSO_4_); ♥—ettringite).

**Figure 9:**
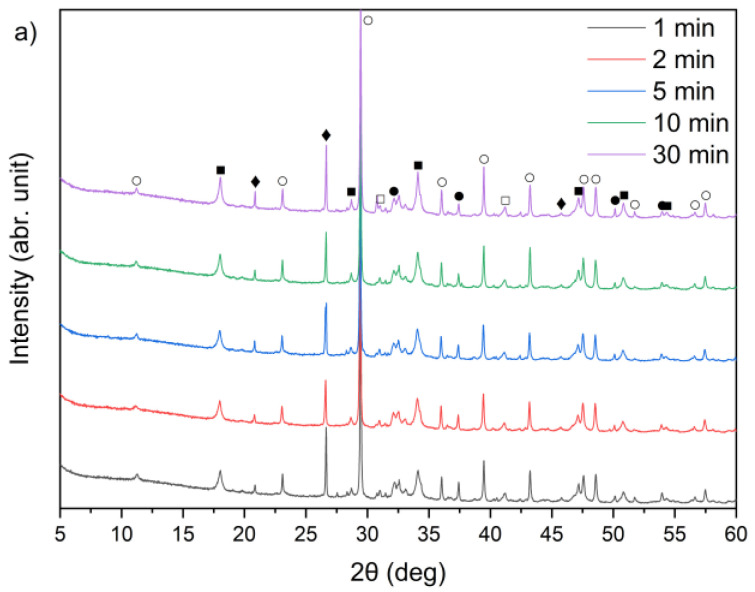

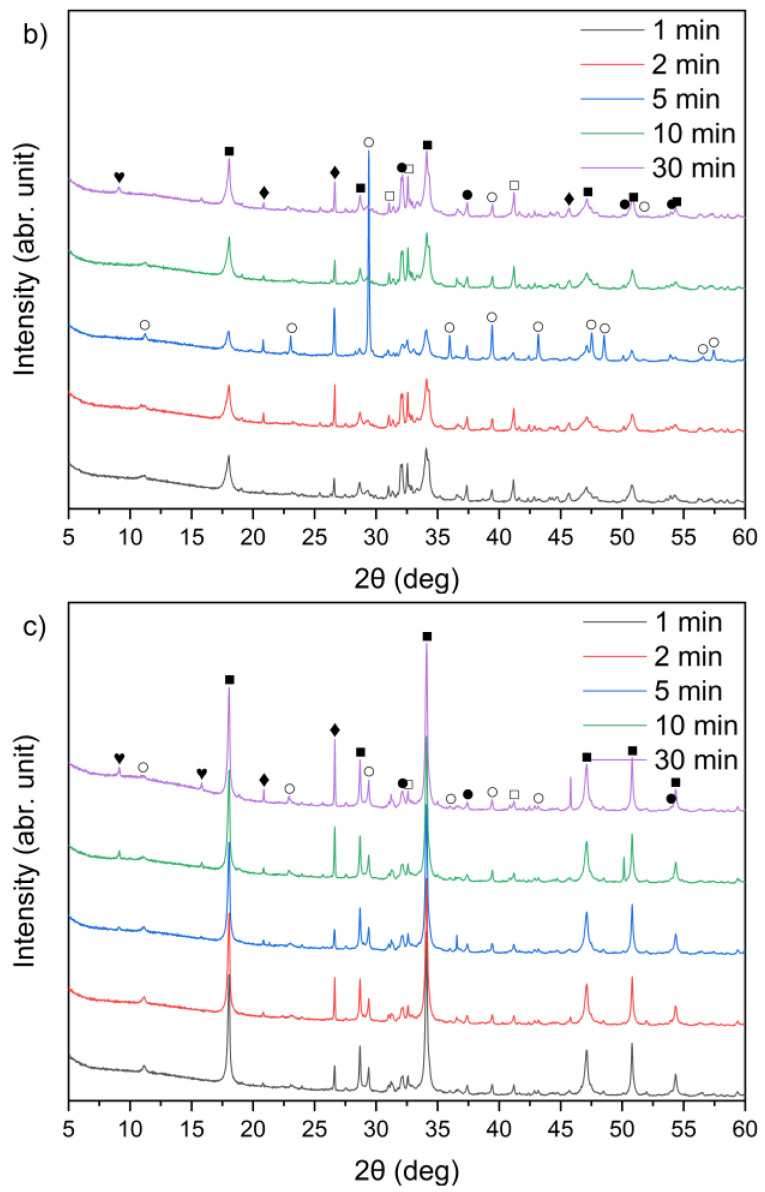
X-ray diffraction patterns of solid leaching residues for (**a**) sample A, (**b**) sample B, and (**c**) sample C at different times (●—lime (CaO); ○—calcite (CaCO_3_); ■—portlandite (Ca(OH)_2_); □—alite (Ca_3_SiO_5_); ♦—quartz (SiO_2_); ♥—ettringite).

The XRD patterns of the solid leaching residues obtained at different temperatures and leaching times are illustrated in Figure 8a–c and Figure 9a–c, respectively. It was observed that the phase composition of samples A, B and C changed significantly after treatment with water. Before the leaching process, lime (CaO) and sylvite (KCl) were identified as the main minerals. According to the XRD analysis presented in Figure 8, after 30 min of leaching, the intensity of sylvite peaks decreased markedly with increasing temperature, and at the highest temperature (90 °C) sylvite diffraction peaks completely disappeared from the solid residues. Similar behavior was observed for the other leaching durations. This indicates that KCl was effectively dissolved during leaching, particularly at higher temperatures. The results also revealed a significant reduction in the intensity of lime (CaO) peaks, accompanied by the emergence of distinct diffraction peaks of portlandite (Ca(OH)_2_). This indicates that CaO underwent hydration upon contact with water, forming calcium hydroxide Ca(OH)_2_. Calcium carbonate (CaCO_3_) was observed as well, likely resulting from the carbonation of Ca(OH)_2_ upon exposure to atmospheric CO_2_.

From the XRD patterns, it can be observed that quartz (SiO_2_) identified in the raw materials (Figure 1) remained largely undissolved in all the solid residues throughout the leaching process. With increasing temperature from 21 °C to 90 °C, a slight decrease in quartz peak intensity was observed. A small peak in the region corresponding to alite (CaSiO_4_) is visible; however, it may also correspond to β- or γ-belite, as alite rapidly hydrates and its presence in the leached residues is uncertain.

An interesting observation was the appearance of the ettringite diffraction peak in samples B and C when the leaching temperature was 21 °C, which diminished as the temperature increased to 45 °C and 90 °C. This is consistent with findings reported by Taylor et al. [52] and Zhou et al. [53], who noted that ettringite is thermally unstable above 70 °C and decomposes into hemihydrate (CaSO_4_⋅0.5H_2_O), water, and monosulfate. A distinct anhydrite (CaSO_4_) peak was detected only in sample B at 90 °C (Figure 8b), suggesting the thermal decomposition of sulfate-bearing phases at higher temperatures.

The identification of hydration and carbonation products, such as portlandite (Ca(OH)_2_) and ettringite, has important implications for the reuse potential of CBPD residues in cement-based materials. The conversion of free CaO to Ca(OH)_2_ increases the alkalinity and reactivity of the material, which may enhance its compatibility with cementitious systems by providing additional sources of hydroxide ions [54,55]. In contrast, excessive Ca(OH)_2_ may lead to overly rapid hydration or incomplete reactions, resulting in a more fragile structure and a decline in compressive strength. Thus, it would be essential to investigate an optimal amount of calcium rich—contained solid residue after extraction of by-pass dust as an addition to cement [56]. At the same time, the presence of ettringite indicates the availability of reactive alumina and sulfate, which could contribute to early-age strength development in blended binders [57]. However, excessive formation of such hydration products may also reduce long-term stability due to late crystallization of ettringite and cracking in the concrete structures [58] if not properly controlled. Therefore, understanding these transformations and modulating the CaO/Al_2_O_3_ and SiO_2_/Al_2_O_3_ ratios is crucial for evaluating the suitability of CBPD residues as supplementary cementitious materials or as precursors in alkali-activated systems [59].

By comparing Figure 1 and Figure 9a–c, it can be observed that the characteristic peaks of sylvite disappeared in samples A, B, and C within the first minute of the leaching process, confirming the rapid dissolution of chloride salts. In addition, the intense peaks originally present in the raw bypass dust at diffraction angles of approximately 32–33°, 37–38° and 54–55° (2θ), corresponding to lime (CaO), showed a substantial decrease after just 1 min of contact with water. Concurrently, a new diffraction peak emerged, indicating the formation of portlandite (Ca(OH)_2_) as a result of CaO hydration.

A leaching time extension up to 30 min resulted in ettringite formation in samples B and C (peak in the 9–10° (2θ) range) (Figure 9b,c). In contrast, ettringite was not detected in the leaching residues of sample A under the same conditions (Figure 9a), suggesting differences in sulfate availability or pH stability during the reaction. The observed differences in ettringite formation correlate well with the initial SO_3_ content in the samples. Samples A and B exhibited relatively high SO_3_ levels (6.5% and 6.7%, respectively, Table 1), while sample C’s was significantly lower (2.5%). Despite this, ettringite was detected in B and C, but not in A. This could suggest that the sulfate was either less soluble or consumed in alternative reactions, or the pH conditions were less favorable for ettringite precipitation in sample A.

**Figure 10:**
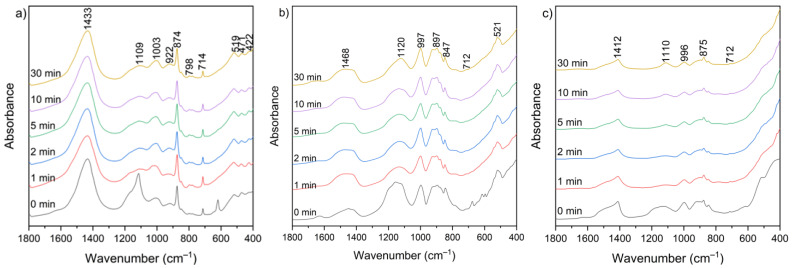
FT-IR spectra of solid leaching residues for (**a**) sample A, (**b**) sample B, and (**c**) sample C at different times.

Figure 10 presents the FT-IR spectra of CBPD samples after water treatment at different leaching times (1–30 min). Several groups of bands in similar wavenumber ranges can be distinguished in each spectrum.

The broad bands in the 3650–3250 cm^−1^ and 1670–1600 cm^−1^ ranges correspond to the existence of hydroxyl groups (–OH) from the reaction of CaO with water and the bending vibration of H–O–H, respectively. These bands appear relatively more intense in the spectra of sample B, both before and after extraction, while they are the weakest in the spectra of sample C after extraction. The range between 1500 and 1400 cm^−1^ corresponds to the presence of stretching vibrations of carbonate groups (C–O) associated with calcium carbonate formation. These bands are clearly more intense in sample A relative to the other bands, which may be related to the higher calcite content observed in the XRD patterns (Figure 9). In the 1300–900 cm^−1^ region, a band near 1100 cm^−1^ can be assigned to sulfate groups (SO_4_^2−^), while the 1050–900 cm^−1^ region corresponds mainly to Si–O–Si(Al) asymmetric stretching vibrations [60]. Additional features in the 800–600 cm^−1^ and 600–400 cm^−1^ ranges are attributed to Si–O–Si(Al) symmetric stretching and bending vibrations, respectively. These bands appear relatively most intense in the spectra of sample B. No major changes in overall functional group composition were observed with varying leaching time. The only noticeable variations were slight increases in the asymmetric stretching vibrations of Si–O–(Si,Al) in the 1300–900 cm^−1^ region, suggesting relative exposure or reorganization of aluminosilicate phases during leaching. The functional group associated with silicates, detected before leaching, was not clearly observed in the post-leaching spectra, likely due to the partial removal of highly soluble phases during the leaching process.

### 3.5. Effect of Leaching Conditions on the Mineralogical Composition and Surface Chemistry

**Figure 11:**
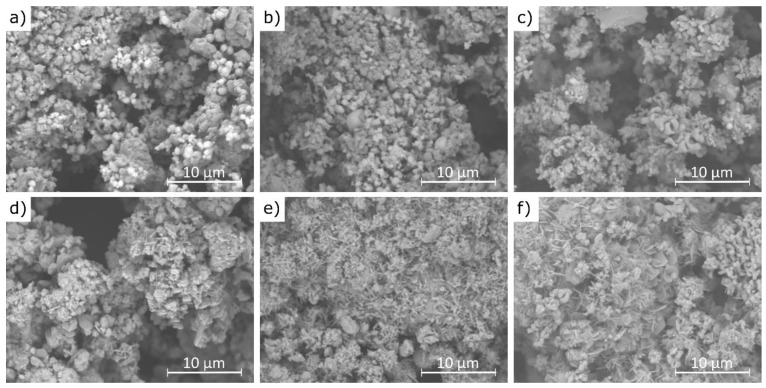
SEM images of CBPDs: (**a**,**d**)—sample A; (**b**,**e**)—sample B; (**c**,**f**)—sample C, images (**a**–**c**)—before extraction; images (**d**–**f**)—after 30 min 30 min of extraction.

Example scanning electron microscopy (SEM) images of cement bypass dust before and after 1 min water extraction at 21 °C are presented in Figure 11.

The results of this study demonstrate that the agglomerates maintain a similar size range (10–20 μm) before and after the extraction process, although their morphology changes noticeably. Figure 11a,b reveal that the pre-leaching microstructure is composed of densely packed sylvite (KCl), visible as bright, well-formed octahedral crystals [61]. These crystals are prominent in samples A and B, while in sample C they occur only sporadically. This observation is fully consistent with the XRF analysis results, which showed the lowest chlorine content in sample C. After the water extraction process, the characteristic octahedral sylvite crystals disappear, indicating their dissolution, while new morphologies emerge. These include plate-like structures attributed to portlandite (Ca(OH)_2_) and needle-like formations identified as ettringite (Ca_6_Al_2_(SO_4_)_3_(OH)_12_·26H_2_O) [62], as seen in Figure 11e,f. The presence of these newly formed phases is supported by the XRD analysis, which confirmed the transformation of CaO into Ca(OH)_2_ and the formation of ettringite in samples B and C. The disappearance of sylvite confirms its high water solubility and rapid leaching behavior, even after just 1 min of contact with water. The transformation of CaO into portlandite results from its hydration in aqueous conditions. The formation of ettringite suggests localized chemical changes during leaching, including the availability of sulfate (SO4)^2−^ and reactive alumina (Al_2_O_3_), particularly in samples B and C, which also exhibited higher SO_3_ contents (6.5% and 6.7%, respectively) compared to sample C (2.5%). This difference in sulfate content helps explain why ettringite formation was not observed in sample A under the same conditions.

## 4. Conclusions

The experiments investigated the influence of leaching time, temperature, and chemical composition on the removal of chloride ions from three types of cement bypass dust (CBPD) immersed in water. The results demonstrated that chlorine can be effectively removed from CBPDs using a water-based extraction method, with efficiencies ranging from 70 to 90%. Further extensions of leaching time or increases in temperature did not improve chlorine removal, while extraction at higher temperatures proved energetically unfavorable. The optimal conditions for chloride leaching were found to be 1 min at approximately 21 °C. After water extraction of chloride, the composition is concentrated, and the content of Ca, Al, Si, Mg, and Fe oxides in the solid residues is increased.

No linear or predictable correlation was observed between the leaching parameters and the extent of metal enrichment in the solid residues. However, general trends were identified, with more pronounced oxide concentration changes in samples with higher initial chloride content. For instance, calcium oxide concentrations increased by approximately 40% in CBPDs with high initial chloride content (13.65% and 15.43%), while for a CBPD with low chloride content (4.32%), the increase was only around 10%.

From a practical perspective, the reuse of dechlorinated CBPD should be considered in the context of existing standards for cement and concrete. According to standards, the content of soluble alkalis and chlorides in cement and concrete must be strictly controlled, as excessive levels can lead to durability issues such as alkali–silica reaction or corrosion of steel reinforcement. The water-leaching process applied in this study significantly reduced the chloride content of CBPD, bringing it closer to the threshold values defined in the standard. This suggests that the treated material could be reused as a minor additional constituent in cement, concrete, or partially substituted as a raw material in clinker production, without compromising compliance with EN 197-1 and EN 206: 2013 requirements. The findings of this research may contribute to increasing the utilization rate of the CBPD as a supplementary mineral additive in cement and concrete production. However, further research should focus on practical case studies and explore the impact of dechlorinated CBPD on the mechanical and durability properties of cement-based materials to fully validate that strength or other performance criteria are met.

## Figures and Tables

**Table 1:** Oxide content in dust from chlorine bypass installations.

Sample	SiO_2_	Al_2_O_3_	Fe_2_O_3_	CaO	MgO	Na_2_O	K_2_O	SO_3_	LOI	Cl
A	8.03	2.52	1.94	49.47	0.69	1.02	14.91	6.51	12.97	13.65
B	7.19	2.44	2.00	49.53	0.62	4.42	10.21	6.71	10.12	15.43
C	7.12	2.16	2.35	73.64	1.22	0.73	4.52	2.49	7.35	4.32

**Table 2:** Oxide content in dust from chlorine bypass installations [45,46].

Constitute	Concrete Composition, kg/m^3^	Chloride Content, %	Total Chloride Content, kg
Cement	315	0.08	0.2520
Fine Aggregate	790	0.04	0.3160
Coarse Aggregate	1080	0.01	0.1080
Admixture	1	0.10	0.0010
Solid residue (CBPD)	10	0.50	0.0175
Water	160	0.00	0.0000
Total	2381	0.28	0.6945

## Data Availability

The original contributions presented in this study are included in the article. Further inquiries can be directed to the corresponding author.
